# Radiomics Analysis of QUS Spectral Parametric Images for Predicting the Risk of Breast Cancer Recurrence

**DOI:** 10.3390/cancers17233810

**Published:** 2025-11-28

**Authors:** Laurentius Oscar Osapoetra, Graham Dinniwell, Maria Lourdes Anzola Pena, David Alberico, Lakshmanan Sannachi, Gregory J. Czarnota

**Affiliations:** 1 Physical Sciences, Sunnybrook Research Institute, Sunnybrook Health Sciences Centre, Toronto, ON M4N 3M5, Canada; laurentiusoscar.osapoetra@sunnybrook.ca (L.O.O.); graham.dinniwell@gmail.com (G.D.); marialourdes.anzolapena@sri.utoronto.ca (M.L.A.P.); david.alberico@sri.utoronto.ca (D.A.); lakshmanan.sannachi@sunnybrook.ca (L.S.); 2 Department of Radiation Oncology, Odette Cancer Centre, Sunnybrook Health Sciences Centre, Toronto, ON M4N 3M5, Canada; 3 Department of Radiation Oncology, University of Toronto, Toronto, ON M5T 1P5, Canada; 4 Department of Medical Biophysics, University of Toronto, Toronto, ON M4N 3M5, Canada

**Keywords:** Oncotype DX, breast cancer recurrence, QUS spectral parametric imaging, radiomics, machine learning

## Abstract

**Background/Objectives:** To evaluate the ability of radiomics analysis of QUS spectral parametric imaging to non-invasively differentiate intermediate-to-high-risk from low-risk Oncotype DX^TM^ Recurrence Score (ODXRS). **Methods:** This prospective study included 31 participants (21 intermediate-to-high-risk ODXRS (median age, 56 years [IQR: 49–68 years]) and 10 low-risk ODXRS (median age, 52 years [IQR: 48–58 years])) presenting with ER+ HER2− invasive breast masses acquired between September 2015 and August 2024. Quantitative ultrasound (QUS) spectroscopy produced five spectral maps, from which radiomics features (including statistical, texture, and morphological measures) were extracted from the tumor core and a 5 mm margin. The ground truth label was determined from thresholding the ODXRS. A multivariate predictive model was developed to differentiate intermediate-to-high-risk ODXRS from low-risk ODXRS, with performance assessed via nested leave-one-out cross-validation (LOOCV). **Results:** A nested leave-one-out cross-validation (LOOCV) analysis demonstrated the generalization performance of a four-feature model. The support vector machine (SVM-RBF) classifier achieved 86% recall, 100% specificity, 93% balanced accuracy, and an area under the receiver operating characteristic curve (AUROC) of 0.95 (CI = 0.88–1.00) in identifying intermediate-to-high-risk versus low-risk ODXRS. **Conclusions:** The preliminary results suggest the potential radiomics-based model of ODXRS in predicting the risks of recurrence. The results warrant further investigation on a larger cohort. This framework can be a useful surrogate for participants for whom ODX testing is neither affordable nor available.

## Simple Summary

The Oncotype DX^TM^ Recurrence Score (ODXRS) is a well-established assay to predict the likelihood of distant breast cancer recurrence and determine the potential benefit of adjuvant chemotherapy. However, its high cost limits accessibility. To address this, we propose a cost-effective, imaging-based multivariate predictive model that leverages radiomics features extracted from QUS spectral parametric images of malignant breast tumors. The model’s generalizability was validated through nested cross-validation analysis, demonstrating its potential as an alternative US imaging-based tool for recurrence risk assessment.

## 1. Introduction

The Onctoype DX^TM^ Recurrence Score (ODXRS) is a 21-gene assay used to estimate the risk of distant recurrence and to evaluate the potential benefit of adjuvant chemotherapy for a specific subtype of breast cancer with hormone receptor-positive (HR+), human epidermal growth factor receptor 2-negative (HER2−), and lymph node-negative (LN−) early-stage invasive breast cancer [1,2,3]. The recurrence score algorithm incorporates genes associated with tumor cell proliferation and hormonal response [1,2,3]. These genes have been reported to correlate with chemotherapy response.

Several clinical studies have validated the prognostic and predictive utility of ODXRS in this patient population [1,2,4,5]. Notably, the National Surgical Adjuvant Breast and Bowel Project (NSABP) Protocol B-14 and B-20 trials retrospectively demonstrated a significant improvement in 10-year distant recurrence-free survival (DRFS) when chemotherapy was added to tamoxifen in patients with high-risk ODXRS [1,2]. Furthermore, the Trial Assigning Individualized Options for Treatment (TAILORx) prospectively confirmed that adding chemotherapy improved 9-year DRFS in patients with high-risk ODXRS across all age groups, as well as in patients with intermediate-to-high-risk ODXRS aged 50 years or younger [4,5]. ODXRS also supports risk stratification to guide the selective omission of radiotherapy (RT) in low-risk patients undergoing breast-conserving therapy [6,7]. Despite its proven clinical utility and commercial availability, the assay remains prohibitively expensive for routine use [8]. As a cost-effective alternative, imaging-based models capable of accurately predicting ODXRS risks may offer a more affordable solution for patient management.

Quantitative US (QUS) techniques extract various tissue microstructural characteristics that are believed to carry diagnostic and prognostic significance, overcoming the limitations of conventional B-mode imaging. For example, QUS spectroscopy analyzes the spectral content of US radiofrequency (RF) data to study acoustic backscattering properties. The technique has been widely applied in several areas, including characterizing tumors [9,10,11], monitoring tumor response to cancer treatments [12,13,14], and detecting tumor deposits in ex vivo lymph nodes [15]. In contrast, QUS echo envelope analysis uses statistical methods to examine the spatial arrangement of acoustic scatterers from the envelopes of US RF signals [16,17]. Additionally, US elasticity imaging, including shear wave elasticity imaging (SWE), provides macro-elasticity parameters, such as shear modulus, for tumor characterization [10].

Radiomics is a rapidly evolving field in medicine and oncology that emphasizes the extraction of quantitative features from medical images. While imaging has traditionally served a diagnostic role, advances in computer vision have enabled the development of non-invasive imaging biomarkers [18,19]. This high-dimensional image analysis can be applied across various imaging modalities such as US, CT, MRI, and PET, all commonly used at different stages of cancer management. The quantitative features derived from these imaging techniques have shown associations with a wide range of clinical endpoints, including histopathologic and molecular profiling, prognostication, and assessment of treatment response [20]. These features typically include first-order statistics, second-order texture patterns, and morphological attributes [21]. When integrated with advanced machine learning and deep learning techniques, radiomics provides a powerful imaging-based framework to support and enhance cancer care.

Previous studies have applied radiomics analysis to predict the ODXRS using regression models [22,23] or to classify ODXRS categories using classification models [24,25,26,27,28]. However, these studies primarily utilized MRI [24,25,26,27,29,30] and mammography images [28]. In contrast, our study seeks to evaluate the effectiveness of radiomics analysis applied to QUS spectral parametric images for ODXRS classification. QUS Spectral parametric images, which portray the spatial distribution of scattering properties within tumor microstructures, offer a unique opportunity for quantitative analysis in both prognosis [12,13,14] and diagnosis purposes [9,10,11]. Furthermore, tumor heterogeneity assessment, as quantified through radiomics textural features of QUS spectral parametric images, provides a means for prognostic assessment.

We hypothesized that radiomics signatures from QUS spectral parametric images can be used to develop a robust multivariate model that offers a surrogate prediction for distinguishing intermediate-to-high-risk ODXRS from low-risk ODXRS, in a cohort of ER-positive (ER+), human epidermal growth factor receptor 2-negative (HER2−), lymph node negative (LN−) invasive breast cancers. These models could serve as valuable imaging tools to assist oncologists and patients in evaluating the cost-effectiveness of ODX testing, as well as providing alternative predictions when ODX testing is neither affordable nor accessible. This could serve as a valuable tool for individualized treatment planning, particularly in guiding adjuvant therapy recommendations.

## 2. Materials and Methods

### 2.1. Participant Selection

The institutional research ethics board (SUN-2094) approved this prospective study conducted in a single institution and registered with clinicaltrials.gov (NCT04050423). The study was conducted following good clinical practice according to the Declaration of Helsinki. All participants provided written informed consents for their participation. Study accrual was carried out from September 2015 to date, whereas the current analysis utilizes participant image data acquired between September 2015 and August 2024.

A clinical prognosis task for breast cancer recurrence was addressed, identifying participants with intermediate-to-high-risk Oncotype DX^TM^ Recurrence Score (ODXRS) from those with low-risk ODXRS, using radiomics characterization of QUS spectral parametric images. We hypothesized that radiomics features derived from QUS spectral parametric maps could be used to develop a robust predictive model to discriminate medium- or high-risk from low-risk ODXRS. To tackle this problem, we formulated a predictive analytics approach that builds a multivariate model. We selected an ODXRS threshold of 15 to differentiate between the two groups. This threshold was selected taking into account the previously used thresholds for low-risk ODXRS from the NSABP [1,2] and the TAILORx clinical trials [4,5], where recurrence scores below 18 [1,2] and less than or equal to 10 [4,5] were utilized, respectively.

The cohort consisted of *n* = 31 participants, with 10 having low-risk ODXRS (≤15) and 21 having intermediate-to-high-risk ODXRS. This represents a small subset of the malignant breast cancer participants from the larger breast imaging study at Sunnybrook, where the ODXRS are available. The inclusion criteria included (1) histologically or cytologically confirmed breast carcinoma (breast or Axilla), stages I–IV, (2) measurable breast or axilla disease by US or MRI, performed within 28 days prior to treatment, (3) ODX testing, and (4) hormone receptor-positive (ER+, PR+), HER2−, and LN− malignant breast cancer participants. All participants provided written informed consent. A total of 7 participants were excluded from the initial sample of 38, resulting in a final cohort of 31 participants. The exclusion criteria included the absence of quantitative ODXRS (*n* = 2), non-standard US RF acquisition settings (*n* = 2), missing US RF data (*n* = 2), and a non-sonographically identified mass (*n* = 1).

### 2.2. Data Acquisition

US RF data was collected using a clinical US system capable of acquiring RF signals. Specifically, a Sonix Touch US imaging system (Ultrasonix Medical Corp., Richmond, BC, Canada) was used, equipped with a linear-array transducer (L14-5/60W) operating at a center frequency of 6.5 MHz and a bandwidth of 3–8 MHz. Table A1 details the characteristics of the US imaging system used.

A radiologist or sonographer experienced in breast US imaging conducted the scans and manually delineated the tumors along with their 5 mm tumor rims. Multiple US RF frames were collected across the three-dimensional tumor volume, representing slices from the tumors. Subsequently, parametric maps from the regions of interest (ROIs) were created (described below), where radiomics features were subsequently extracted.

### 2.3. QUS Spectral Parametric Imaging

In addition to obtaining standard B-mode images of tumors, QUS spectral parametric images for both tumors and their 5 mm margins were calculated. These QUS spectral parameters were derived from the spectral analysis of windowed US RF data. The approach involved employing a 2 mm by 2 mm sliding window with a 94% window overlap in both the range and lateral directions. The window size was selected to encompass an adequate number of acoustic wavelengths for reliable spectrum estimation while preserving the resolution necessary for distinguishing areas of distinct microstructures [31]. This window was systematically moved across all points within the ROI to capture each pixel in the resulting parametric images.

In order to estimate the average spectrum from a block of US RF data, a Hanning gating function was applied along the range direction to the RF signal. Subsequently, a fast Fourier transform algorithm was utilized to transform the RF signal into its frequency domain representation. As a next step, the average power spectra across the lateral directions (columns in the RF block) were computed to derive an averaged power spectrum. Additionally, an attenuation correction was incorporated to account for US attenuation caused by the propagation through intervening tissue layers and the tumor. For the intervening skin, a predetermined attenuation coefficient value was assumed, while the attenuation coefficient of the tumor was estimated using a spectral difference method [32].

We obtained spectral parameters by parametrizing the averaged power spectra. Linear parametrization resulted in linear-fit spectral parameters, including mid-band fit (MBF), spectral slope (SS), and 0 MHz spectral intercept (SI). MBF and SI are indicators of the amount of acoustic backscattering, whereas SS is associated with the effective size of acoustic scatterers [31]. Given that these measurements offer somewhat indirect physical information about the acoustic scatterers, an acoustic scattering model was applied that leveraged a spherical Gaussian form factor to fit the measured backscatter coefficient (BSC) from the tissues. This analysis enabled the estimation of properties related to the scatterers, encompassing average acoustic concentration (AAC) and average scatterer diameter (ASD) parameters [31].

The analysis encompassed all points within the ROI, resulting in parametric images that depicted the spatial distributions of QUS spectral parameters. It was hypothesized that these parametric images, serving as surrogates for acoustic microstructures, could be leveraged to differentiate intermediate-to-high-risk ODXRS from low-risk ODXRS. Subsequently, various numerical characteristics were extracted related to first-order statistics of pixels and texture from these images. In addition, morphological features from the two-dimensional regions of interest were also extracted, along with radiomics features.

### 2.4. Feature Engineering

Radiomics features were determined from the parametric images of the breast tumor and its 5 mm tumor rim. The open-source software package PyRadiomics (version 3.1.0) was utilized to obtain these radiomics features [21], allowing for standardization of the process.

Prior to extracting features, we normalized the pixels and applied a scaling of 100. Outlier pixels were removed at a cut-off of ±3 standard deviations. Subsequently, we resampled the parametric maps into a uniform grid of 0.12 mm × 0.12 mm using sitkBSpline interpolator from PyRadiomics (version 3.1.0). This spacing corresponds to the lateral spacing of the parametric map. For feature extraction, the resampled pixels were quantized using a fixed bin width of 15. Features were extracted from the average of neighbors’ distances of 1, 2, 3, 4, and 5. Feature extraction settings for the wavelet features followed the PyRadiomics (version 3.1.0) default settings. Particularly, the type of wavelet used for the decomposition was ‘coiflets’ (Coif1). The wavelet decomposition started from the original image and proceeded up to the first level, resulting in a set of wavelet decompositions {‘LL’, ‘LH’, ‘HL’, and ‘HH’}, where ‘L’ refers to a low-pass filter and ‘H’ refers to a high-pass filter.

A total of 4659 two-dimensional features were computed from QUS spectral parametric images of the tumor core and its margin. These include the first-order statistical features (*n* = 18), two-dimensional shape or morphological features (*n* = 9), and textural features. First-order statistical features describe the distribution of voxel intensities within the region of interest (ROI) utilizing commonly employed basic metrics [21]. Two-dimensional shape features provide descriptors of the size and shape of the ROI [21]. Textural features quantify the spatial arrangement of voxels, offering estimates such as coarseness, contrast, and regularity. These include gray-level co-occurrence matrix (GLCM) features (*n* = 24) [33], gray-level run length matrix (GRLM) features (*n* = 16) [34,35,36,37], Gray-Level Size Zone Matrix (GLSZM) features (*n* = 16) [38], Neighboring Gray Tone Difference Matrix (NGTDM) features (*n* = 5) [39], and gray-level dependence matrix (GLDM) features (*n* = 14) [40]. The textural features were extracted from both the original and wavelet-filtered parametric maps. Table A2 tabulates the different features extracted.

For each patient, vectors of radiomics features were obtained from parametric maps, and subsequently averaged based on the ROI size to obtain the weighted, averaged vector of radiomics features. These vectors of radiomics features constitute the data matrix.

We formulated a predictive analytics problem to develop a multivariate radiomics model for predicting intermediate-to-high-risk vs. low-risk ODXRS. We selected an Oncotype DX score threshold of 15 between the two groups. The dataset consisted of *n* = 31 malignant breast lesions, with 21 participants with intermediate-to-high-risk ODXRS and 10 participants with low-risk ODXRS.

### 2.5. Data Preprocessing

#### 2.5.1. Data Partitioning

**Figure 1:**
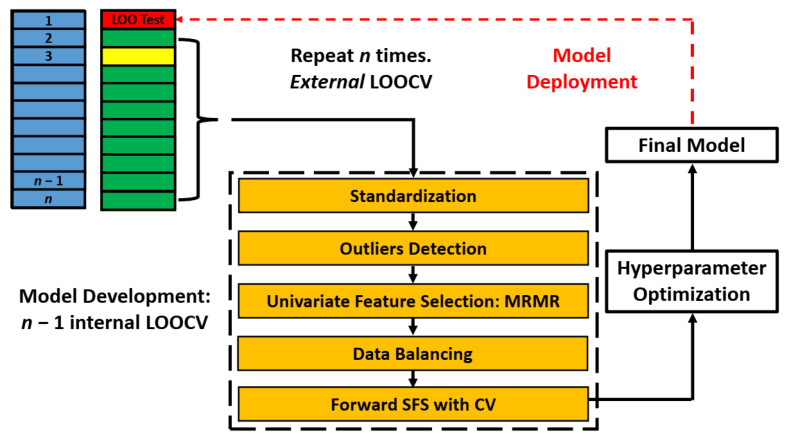
Model Building and Evaluation Schematic: Nested leave-one-out cross-validation for model building and evaluation. The data were split into *n* development-test folds, each consisting of *n* − 1 samples for model development (green- and yellow-shaded) and a single sample for testing (red-shaded). Within each development fold, an internal LOOCV was performed on the *n* − 1 samples to conduct feature selection and optimize hyperparameters. Model fitting was performed on the *n* – 2 samples (green-shaded), while model validation utilized a LOO sample (yellow-shaded). The features and hyperparameters that achieved the best average performance on the internal validation samples were selected. A final model was then trained on the entire *n* − 1 development samples using the selected features and optimized hyperparameters, and evaluated on the held-out test sample. The prediction scores from all *n* LOO test samples were thresholded and aggregated to construct a confusion matrix, from which classification metrics were derived. SFS: Sequential feature selection. LOOCV: Leave-one-out cross-validation.

We implemented a nested leave-one-out cross-validation (LOOCV) for model building and evaluation. We created *n* folds for development and test sets. In each fold, we left out one sample and developed a model with the remaining *n* − 1 samples. We created internal cross-validation partitions using the *n* − 1 samples for sequential feature selection and hyperparameter optimization. The final model was then fitted on the *n* − 1 samples and tested on the leave-one-out sample. The process is repeated with *n* different models trained and prediction scores obtained from leave-one-out samples. Figure 1 illustrates the approach.

#### 2.5.2. Standardization and Outlier Identification

For the development set, we applied feature standardization by subtracting the mean and dividing by the standard deviation. We identified outliers using the Isolation Forest technique, assuming a 5% contamination rate [41].

#### 2.5.3. Feature Selection/Dimension Reduction

We performed dimensionality reduction with filter-based feature selection, based on the Maximal Relevance Minimal Redundancy (MRMR) criterion [42], to identify 50 features with the highest relevance to the target class and the least redundancy. We adopted two approaches to address data imbalance: data-based and algorithmic-based methods. For classifiers that do not support assigning different class weights, we balanced the development set using the synthetic minority oversampling (SMOTE) technique [43]. In contrast, for classifiers that support class weighting, we assigned appropriate weights to the different classes. Subsequently, we utilized wrapper-based feature selection using a forward sequential feature selection (SFS) method to identify the optimum four features, based on the balanced accuracy metric.

### 2.6. Model Building and Evaluation

Data preprocessing, model building, and evaluation were performed using an open-source Scikit-Learn software package (version 1.7.2). We fitted several machine learning classifiers, including linear discriminant analysis (LDA), *k*-nearest neighbors (KNN), linear support vector machines (SVM), and Random Forest (RF). We developed *n* separate models using *n* − 1 samples and each time predicting the left-out sample. The prediction probabilities for each leave-one-out sample were aggregated to create a final test set confusion matrix. Internal *n* – 1 LOOCV was employed to select the optimal model, including its hyperparameters. We performed an exhaustive grid search cross-validation to search for the optimum set of hyperparameters. Table A3 lists the hyperparameters for the different classifiers evaluated. The optimum set of hyperparameters was selected based on the best validation balanced accuracy metric, utilizing the *n* – 1 internal LOOCV. Furthermore, we performed model complexity analysis by varying the number of selected features from three to seven features and evaluating the generalization performance of the models. The range of features was determined by the size of the cohort to reduce the risk of overfitting.

We reported the classification performance on the leave-one-out test set, including recall, specificity, accuracy, balanced accuracy, precision/positive predictive value (PPV), negative predictive value (NPV), *F*1-*Score*, area under the receiver operating characteristic curve (AUROC) metrics, and area under the precision–recall curve (AUPRC). The metrics are defined as follows:

$$\mathrm{Recall}\;=\;\frac{TP}{TP\;+\;FN}$$

$$\mathrm{Specificity}=\frac{TN}{TN+FP}$$

$$\mathrm{Accuracy}=\frac{TP+TN}{TP+FN+TN+FP}$$

$$\mathrm{Balanced}\;\mathrm{Accuracy}=\frac{\left(Recall+Specificity\right)}{2}$$

$$\mathrm{Precision}=\frac{TP}{TP+FP}$$

$$\mathrm{NPV}=\frac{TN}{TN+FN}$$

$$F1\text{-}\mathrm{Score}=\frac{2\times Precision\times Recall}{Precision+Recall}$$

where TP = true positives, FN = false negatives, TN = true negatives, and FP = false positives.

### 2.7. Statistical Analysis

Statistical analysis was performed using an open-source SciPy software package (version 1.14.0). We test for any statistically significant difference using either a two-sample *t*-test or a Mann–Whitney U-test, depending on the data distribution. In order to assess normality, we employed the Shapiro–Wilk normality test. To counteract the increased risk of false positives resulting from multiple hypothesis tests, a Bonferroni correction was applied to adjust the significance threshold to *α* = 0.05/(# of hypothesis tests). Consequently, each feature was assessed at an adjusted significance level of *α* = 0.05/*n* = 0.001 (*n* = 50-the number of MRMR selected features), ensuring a probability of less than 5% for obtaining one or more false positives.

## 3. Results

### 3.1. Patient Characteristics

The characteristics of the participants involved in this study are summarized in Table 1. All participants were female. A total of 31 participants were included in the study, with 21 intermediate-to-high-risk ODXRS (median age 56 [IQR: 49–68] years) and 10 low-risk ODXRS (median age 52 [IQR: 48–58] years). A total of 68% of patients had invasive ductal carcinoma (IDC), 13% had invasive lobular carcinoma (ILC), and 13% had ductal carcinoma in situ (DCIS). Further, 23% of patients had a grade I tumor, 58% had a grade II tumor, and 19% had a grade III tumor. All participants exhibit ER+, PR+, and HER2−. The tumor mass size was measured along the longest axis; the mean length was 2.2 cm, and the range spanned from 0.7 cm to 8.9 cm.

### 3.2. QUS Spectral Parametric Images

**Figure 2:**
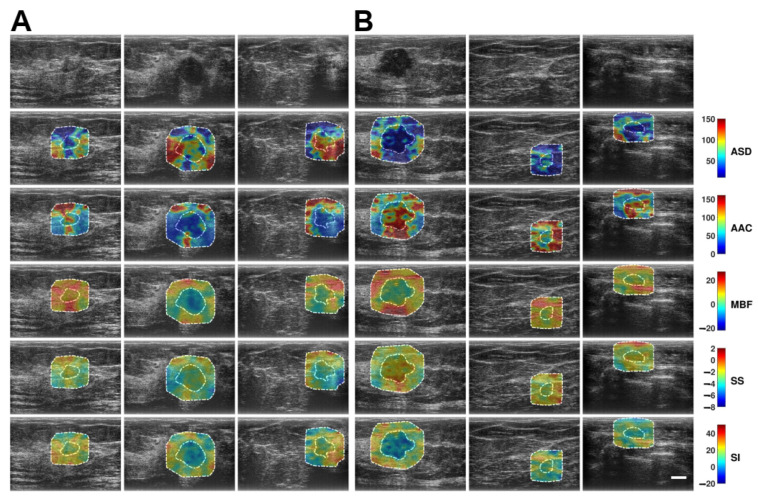
Representative B-mode and QUS spectral parametric images of ASD, AAC, MBF, SS, and SI for (**A**) low-risk Oncotype DX Recurrence Score and ODXRS (left three columns), and (**B**) intermediate-to-high-risk ODXRS (right three columns) malignant breast lesions. The color bar range is 140 µm for ASD, 160 dB/cm^3^ for AAC, 49 dB for MBF, 12 dB/MHz for SS, and 70 dB for SI. The scale bar represents 1 cm. This corresponds to the full FOV of 4 cm axially and 6 cm laterally. The low-risk ODXRS lesions were diagnosed as DCIS, ILC, and IDC, respectively. The intermediate-to-high ODXRS lesions were all diagnosed as IDCs. The comprehensive set of radiomics features, including basic statistical, various textural, and morphological features, was extracted from the tumor core (inner dashed contour) and its 5 mm tumor margin (outer dashed contour). These are utilized to build a multivariate predictive analytics model to differentiate intermediate-to-high risk ODXRS from low-risk ODXRS malignant breast lesions. ASD: Average scattering diameter. FOV: Field-of-view. AAC: Average acoustic concentration. DCIS: Ductal carcinoma in situ. MBF: Mid-band fit. ILC: Invasive lobular carcinoma. SS: Spectral slope. IDC: Invasive ductal carcinoma. SI: Spectral intercept.

Figure 2 shows representative B-mode and QUS spectral parametric images of malignant breast tumors with low-risk ODXRS (A, left three columns) and intermediate-to-high-risk ODXRS (B, right three columns). Although visual discrimination between low and intermediate-to-high risk groups is not immediately perceptible from the images, further quantitative analysis demonstrates discrimination, especially for the features derived from the wavelet-filtered version of the original QUS maps.

### 3.3. Feature Analysis

The implementation of nested LOOCV for model development and evaluation requires building *n* separate models, each trained on a different combination of *n* − 1 samples. This process often results in a different subset of features being selected for the optimal model in each iteration. Here, we present the results from one representative partition. For this partition, nine radiomics features demonstrated statistically significant differences (*p*-values < 0.05) between the low-risk ODXRS and intermediate-to-high-risk ODXRS groups. This feature set includes the GLCM correlation of the ‘LL’-wavelet margin of the AAC (*p*-value = 0.00002), the GLCM correlation of the margin of the AAC (*p*-value = 0.00002), the NGTDM contrast of the ‘LL’-wavelet margin of the SI (*p*-value = 0.001), the GLCM maximal correlation coefficient (MCC) of the margin of the AAC (*p*-value = 0.01), the GLDM Small Dependence Emphasis of the ‘HL’-wavelet margin of the SS (*p*-value = 0.02), the first-order mean absolute deviation of the ‘HH’-wavelet margin of the SI *(p*-value = 0.02), the NGTDM complexity of the ‘LH’-wavelet margin of the SS (*p*-value = 0.03), the GLRLM short-run Low Gray-Level Emphasis of the ‘HH’-wavelet margin of the SI *(p*-value = 0.04), and the first-order minimum of the ‘HH’-wavelet margin of the SS maps *(p*-value = 0.04). Importantly, across all outer loops of the nested LOOCV, features with statistically significant differences were persistently identified.

Accounting for multiple hypothesis tests with a Bonferroni correction, the wavelet-LL GLCM correlation margin AAC and original GLCM correlation margin AAC demonstrated statistically significant differences between intermediate-to-high-risk ODXRS and low-risk ODXRS (*p*-values < 0.001). Although univariate statistical analysis indicated that two radiomic features are statistically significantly different between intermediate-to-high-risk ODXRS and low-risk ODXRS, the combination of several discriminating features can still lead to a generalizable multivariate model.

**Figure 3:**
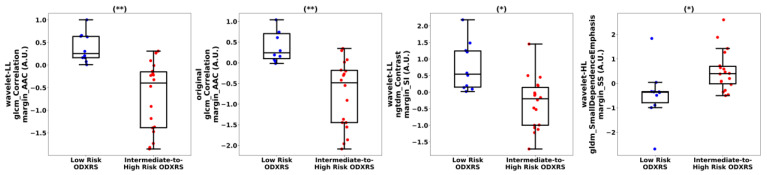
Representative box and scatter plots display discriminative features with statistically significant differences between low-risk ODXRS (blue dots) vs. intermediate-to-high-risk ODXRS (red dots). The presented features were transformed into a standard normal distribution using the *z*-transformation. These features encompass basic statistical, various textural, and morphological features, with the majority being textural and first-order statistical features. Univariate statistical analysis of these features indicated that they are statistically significantly different (*p*-values < 0.05) between low-risk ODXRS and intermediate-to-high-risk ODXRS lesions. A Bonferroni correction was applied to adjust the significance threshold, accounting for multiple hypothesis testing. Features with *p*-values < 0.05 are marked with (*), while those with *p*-values < 0.001 are marked with (**). GLCM: Gray-level co-occurrence matrix. GLRLM: Gray-level run length matrix. Idm: Inverse difference moment. AAC: Average acoustic concentration. ASD: Average scattering diameter. A.U.: Arbitrary unit.

Figure 3 depicts a representation of these features (*p*-values < 0.05). The bottom and top edges of the box represent the 25th and 75th percentiles, respectively. The central mark in each box indicates the median. The whiskers represent 1.5 times the interquartile range.

**Figure 4:**
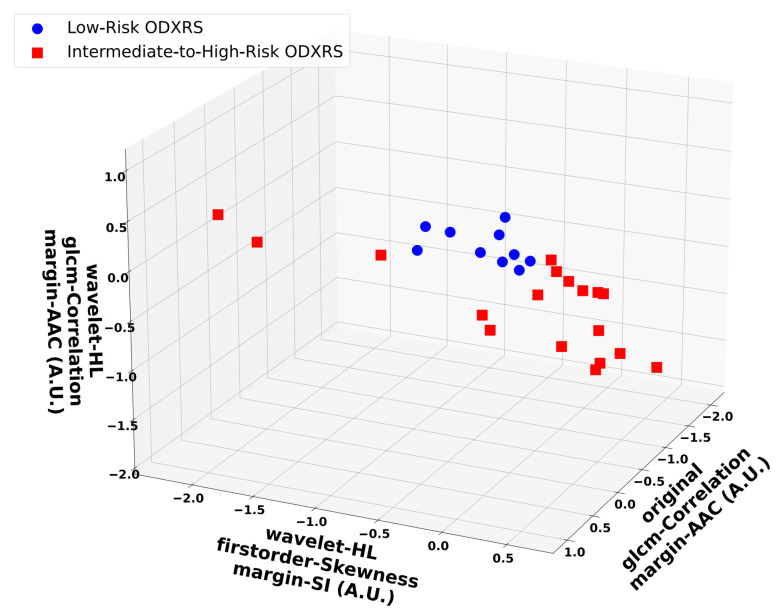
Representative scatter plots for the optimum 4-feature SVM-RBF model. The included features were: GLCM correlation of the margin of the AAC, first-order skewness of the ‘HL’-wavelet margin of the SI, and GLCM Correlation of the ‘LL’-wavelet of the margin of the AAC maps. Blue dots represent low-risk ODXRS, while red squares indicate intermediate-to-high-risk ODXRS. The separation between the two clusters is clearly observed in the three-dimensional input feature space. The four-feature SVM-RBF classifier generated a nonlinear decision boundary (due to the RBF kernel) and demonstrated robust generalization performance, as assessed using nested LOOCV. GLCM: Gray-level co-occurrence matrix. AAC: Average acoustic concentration. SI: Spectral intercept. SVM-RBF: Support vector machine-radial basis function. LOOCV: Leave-one-out cross-validation. A.U.: Arbitrary unit.

Figure 4 depicts representative scatter plots of the most frequently selected features from the four-feature SVM-RBF models across all *n* − 1 outer LOO development folds. For visualization, only three features are shown: the GLCM correlation of the margin of the AAC, the GLCM correlation of the ‘LL’-wavelet margin of the AAC, and the first-order skewness of the ‘HL’-wavelet margin of the SI maps. Clusters corresponding to intermediate-to-high-risk ODXRS (red squares) versus low-risk ODXRS (blue circles) can be observed. Evidently, as presented in the following subsection, the nonlinear SVM-RBF classifier is able to separate these two clusters in the input feature space. The two features that were identified by univariate feature selection to be statistically significantly different (*p*-values < 0.001) were consistently selected by the forward SFS process for the SVM-RBF classifier, across all *n* − 1 outer LOO folds.

### 3.4. Classification Results

Table 2 summarizes the classification performance across the LOO test samples using nested LOOCV. Among the evaluated machine learning classifiers, the SVM-RBF yielded the best generalization performance, achieving 86% recall, 100% specificity, 93% balanced accuracy, and an AUROC of 0.95 (CI: 0.88–1.00). A four-feature model based on the KNN classifier generalized with 71% recall, 70% specificity, 71% balanced accuracy, and 0.78 AUROC (CI: 0.62–0.94) in predicting intermediate-to-high-risk ODXRS from low-risk ODXRS.

## 4. Discussion

The clinical utility of the Onctoype DX^TM^ Recurrence Score (ODXRS), a 21-gene assay, has been well established [1,2,4,5]. However, its use in predicting the risk of distant recurrence and evaluating the potential benefit of adjuvant chemotherapy in patients with hormone receptor-positive (HR+) breast cancer is often limited by its high cost [8]. Imaging-based model that can predict the ODXRS may provide a more affordable alternative. This study investigates the potential of radiomics features derived from QUS spectral parametric images in stratifying the risk of breast cancer recurrence as indicated by the ODXRS. Our results demonstrate that the multivariate imaging-based model achieved 86% recall, 100% specificity, 93% balanced accuracy, and an AUROC of 0.95 (CI = 0.88–1.00) in distinguishing intermediate-to-high-risk ODXRS from low-risk ODXRS, utilizing nested LOOCV. These findings suggest that the proposed radiomics framework warrants further validation in a larger cohort to assess its generalizability. QUS spectral parametric imaging features can be leveraged to build a robust multivariate model capable of discriminating intermediate-to-high-risk vs. low-risk ODXRS. This approach could serve as a valuable alternative in settings where ODX testing is either cost-prohibitive or unavailable. Furthermore, the portability and widespread availability of US-based imaging provide a distinct advantage over other imaging modalities like MRI and CT, making radiomics analysis of QUS spectral parametric images a promising tool for clinical applications.

As correlates of any of the genes measured by the ODXRS assay have been analyzed in histopathological examination, previous research efforts have tried to use histopathologic and clinic-histopathologic variables to predict ODXRS [44,45,46]. Flanagan et al. reported a multivariate linear regression model, consisting of nuclear grade, mitotic count, PR immunohistochemical score, and HER2/neu status, that achieved an R^2^ score of 0.66 [44]. Subsequently, Orucevic et al. developed a nomogram model as a surrogate prediction of the ODXRS test to separate high-risk ODXRS from low-risk ODXRS [45,46]. Their model utilized clinical and histopathologic variables, including age, tumor size, tumor grade, PR status, and lymph-vascular invasion (LVI) [45,46].

Concurrently, research efforts on developing models as surrogate predictions of ODXRS have focused on utilizing radiomics features from MRI and mammography images. Li et al. developed a multivariate model to distinguish intermediate-to-high-risk ODXRS from low-risk ODXRS using radiomics features from dynamic contrast-enhanced (DCE-MR) images in a cohort of 84 participants with invasive breast cancer [24]. They reported an AUROC of 0.76 using the logistic regression classifier and LOOCV analysis in discriminating high-risk ODXRS (*n* = 27) against low-to-intermediate-risk ODXRS (*n* = 57) [24]. Nam et al. reported a radiomics model based on dynamic contrast-enhanced MRI images to distinguish between intermediate-to-high-risk ODXRS (*n* = 22) from low-risk ODXRS (*n* = 45) [29]. They reported a test AUROC of 0.76 based on radiomics features alone, achieved through an LOOCV analysis [29]. Combining with the clinicopathological features resulted in a more robust model with 0.90 test AUROC [29]. Ha et al. leveraged a convolutional neural network (CNN) to categorize ODXRS in a multi-class and binary classification using DCE-MR images from 134 participants with ER+/HER2− invasive breast cancers [26]. In a multi-class setting, the model discriminates low-, intermediate-, and high-risk ODXRS [26]. For the binary classification, the model identifies intermediate-to-high-risk ODXRS from low-risk ODXRS [26]. They reported an overall recall of 60%, a specificity of 90%, an accuracy of 81%, and an AUROC of 0.92 in the three-class prediction [26]. Furthermore, an overall recall of 87%, a specificity of 81%, an accuracy of 84%, and an AUROC of 0.92 were reported for the binary classification [26]. Romeo et al. developed a multivariate model using a set of radiomics features from dynamic contrast-enhanced (DCE) MRI images in a cohort of 248 participants with ER+, HER2− invasive breast cancer [27]. In discriminating high-risk ODXRS against low-to-intermediate-risk ODXRS, they reported a test AUROC of 0.77 (95% CI: 0.56–0.98) [27]. In separating intermediate-to-high-risk against low-risk ODXRS, they reported a lower test AUROC of 0.51 (95% CI: 0.41–0.61) [27]. Recently, Kim et al. reported a systematic review on the current literature on the use of radiomics of breast MRI to predict the ODXRS [30].

Using mammography images, Mao et al. reported a retrospective study that developed a multivariate model using radiomics features to discriminate intermediate-to-high-risk ODXRS from low-risk ODXRS in a multicenter cohort of 304 participants with ER+, LN− invasive breast cancers [28]. They reported that a radiomics model consisting of HaralickCorrelation_angle45_offset7, GLCMEntropy_AllDirection_offset1_SD, and LongRunGreyLevelEmphasis_AllDirection_offset4_SD achieved an AUROC of 0.75 (0.58–0.93) in the external test set [28]. Furthermore, combining clinical risk factors in tumor grade and HER2 status resulted in a model with an AUROC of 0.84 (0.69–0.99) in the external test set [28].

Previous radiomics studies have predominantly focused on imaging features extracted from MR or mammogram images. In this study, we highlight the potential of radiomics features derived from QUS spectral parametric images to develop a robust multivariate model capable of discriminating intermediate-to-high-risk ODXRS from low-risk ODXRS. A four-feature SVM-RBF model demonstrated strong generalization performance, with a recall of 86%, a specificity of 100%, a balanced accuracy of 93%, and an AUROC of 0.95, evaluated using nested LOOCV. These results are comparable to those of similar radiomics studies based on MRI and mammography, in terms of generalization performance. Our work shares similarities with previous studies by Nam et al. [29] and Ha et al. [26], particularly in distinguishing intermediate-to-high-risk ODXRS from low-risk ODXRS. The key difference, however, is that our study utilizes radiomics features from QUS spectral parametric images, whereas prior research relied on MR imaging features [26,29].

In addressing data imbalance for the particular dataset, we found that class weighting produced the best results with SVM classifiers. For other classifiers, which lacked built-in mechanisms to handle class balance, we applied the SMOTE technique [43], a data-based approach to mitigate the imbalance. The algorithm-based approach adjusts the regularization parameter of the SVM classifiers according to the class weights, assigning higher weights to the minority class. In contrast, the data-based approach generates synthetic samples to upsample the minority class. Given the relatively small size of the dataset, the algorithm-based approach is likely to yield better classification performance than the SMOTE technique, as it works directly with the original class distribution without artificially altering it.

Model complexity analysis was performed by varying the number of selected features from three to six. We found that for the particular dataset, the four-feature model was the optimal model, resulting in the best generalization performance using nested LOOCV. Across the development sets, four features that were selected the most often were features from the tumor margin. These include the GLCM correlation of the AAC map, first-order skewness of the ‘HL’-wavelet of the SI map, GLCM correlation of the ‘LL’-wavelet of the AAC map, and GLDM dependence entropy of the ‘HL’-wavelet of the SS map. This indicates that the radiomics features from the tumor margin were an important component in producing the optimum model. These features were predominantly derived from the wavelet decomposition of the original images. Wavelet decomposition breaks down complex image data into various frequency bands, helping to uncover unique features and patterns that may not be immediately apparent in the original image. This process involves sequentially applying low-pass (‘L’) and high-pass (‘H’) filters to extract low-frequency information (‘LL’) and high-frequency details (‘LH’, ‘HL’, and ‘HH’).

Nested LOOCV analysis guarantees model generalization assessment on a limited sample cohort. Our use of nested LOOCV ensures reliable model performance and evaluation on a limited sample cohort, as emphasized by Vabalas et al. [47] and Chalkidou et al. [48]. The implementation of nested LOOCV mitigates the risk of overfitting, as the methodology constructs two levels of LOOCV by leaving one sample out in a vault for external testing, while developing a model on the *n* − 1 samples using an internal LOOCV. These results warrant further investigations on a larger cohort of patients to further confirm the robustness of radiomics characteristics from QUS spectral parametric images in distinguishing intermediate-to-high risk ODXRS from low-risk ODXRS. The framework can be a working tool that provides a surrogate assessment of ODXRS risk when the ODXRS genetic assay is not affordable or not available.

Several limitations of this study include the cohort size and the assessment of feature stability. The small cohort size is a result of the pilot nature of our study. However, we are actively expanding the current cohort and will report on the extended dataset in future publications. As the extracted features are used for prognosis, it is crucial to conduct a feature stability analysis to confirm the robustness of the proposed model. The extracted radiomics features are influenced by the random scattering media of tumor microstructure, the US acquisition system, and the feature extraction software (along with its specific settings). Since such an analysis requires a separate dedicated study, we refer to the work of Sannachi et al., who specifically performed this examination on tumor response assessment for a cohort of locally advanced breast cancer (LABC) patients receiving neo-adjuvant chemotherapy (NAC) [12]. In that study, the effects of different US systems and variations in random media on the reliability of extracted radiomics features from QUS spectral parametric images were examined [12]. They confirmed the reproducibility of the extracted features and concluded that tissue heterogeneity was the dominant source of variability in the measured features, whereas the contribution of US system components was minimal [12]. When repeatability assessments are conducted with immediate successive acquisitions, the random media can be assumed to remain approximately constant. Additionally, since the US system components were fixed across all acquisitions, they did not contribute to variability in the radiomics features. Finally, we used a single software platform with consistent acquisition and processing settings, which supports the notion of reproducibility for the extracted features.

Future work will focus on assessing the robustness of the proposed radiomics framework in discriminating intermediate-to-high risk ODXRS from low-risk ODXRS in a larger patient cohort. Additionally, we aim to differentiate high-risk ODXRS from low-to-intermediate-risk ODXRS, as prior clinical studies have highlighted the importance of identifying the high-risk ODXRS sub-group. Patients in this category are most likely to benefit from adjuvant chemotherapy, which can significantly improve the prognosis for distant recurrence [3]. In the current study, the number of samples in the high-risk ODXRS sub-group is relatively small (*n* = 6), making the stratification of high-risk ODXRS versus low-to-intermediate-risk ODXRS more challenging. Furthermore, expanding the study to a larger cohort study will enable the application of advanced deep learning techniques, such as convolutional neural networks (CNNs) [49] and vision transformers (ViTs) [50], which have demonstrated significant improvements in generalization performance across various classification tasks, particularly in the characterization of breast lesions [51]. This work complements other works on breast cancer [52,53,54].

A QUS-based model capable of accurately predicting ODXRS risk could serve as a valuable and cost-effective tool for individualized treatment planning, particularly in guiding adjuvant therapy recommendations. For example, it could recommend adjuvant therapy for breast cancer patients with a higher likelihood of recurrence who would benefit from adjuvant chemotherapy. This includes women of any age with a high-risk ODXRS (greater than 25) or women 50 years of age or younger with an intermediate-to-high risk ODXRS (15–25) [4,5]. Notably, the ODXRS has demonstrated a significantly greater reduction in the utilization of adjuvant chemotherapy compared to other widely employed multigene assays. This reduction in chemotherapy, along with its associated side effects and increased risk of secondary cancers, may result in substantial cost savings for healthcare systems.

## 5. Conclusions

The QUS spectral parametric imaging radiomics SVM-RBF model demonstrates significant potential as a non-invasive, imaging-based phenotyping approach for the stratification of breast cancer recurrence risk. This model achieves 86% recall, 100% specificity, 93% balanced accuracy, and an AUROC of 0.95 (CI = 0.88–1.00) in distinguishing intermediate-to-high risk ODXRS from low-risk ODXRS, utilizing nested LOOCV. In contrast to previous studies that extracted radiomic features from MRI and mammogram images to build models for stratifying ODXRS risk, our pilot investigation suggests that QUS spectral parametric imaging radiomics could serve as a valuable tool in the decision support systems for adjuvant therapy recommendations in invasive breast cancer.

## Figures and Tables

**Table 1:** Clinical Characteristics: ODXRS cohort clinical characteristics.

Characteristics	Low-Risk ODXRS (*n* = 10)	Intermediate-to-High-Risk ODXRS (*n* = 21)	All (*n* = 31)
Age (y)			
Mean (SD)	54 (8)	57 (12)	56 (11)
Median (Q1, Q3)	52 (48, 58)	56 (49, 68)	55 (48, 63)
Min, max	46, 74	33, 78	33, 78
Tumor size (cm)			
Mean (SD)	2.4 (2.3)	2.1 (1.1)	2.2 (1.6)
Median (Q1, Q3)	1.4 (1.2, 2.1)	1.9 (1.3, 2.8)	1.7 (1.2, 2.8)
Min, max	1.1, 8.9	0.7, 4.6	0.7, 8.9
Invasive tumor type *n* (%)			
Invasive ductal carcinoma	7 (70%)	14 (67%)	21 (68%)
Invasive lobular carcinoma	1 (10%)	3 (14%)	4 (13%)
Ductal carcinoma in situ	1 (10%)	3 (14%)	4 (13%)
Other	1 (10%)	1 (5%)	2 (6%)
Histologic tumor grade, *n* (%)			
Grade I	2 (20%)	5 (24%)	7 (23%)
Grade II	6 (60%)	12 (57%)	18 (58%)
Grade III	2 (20%)	4 (19%)	6 (19%)
Hormone receptor status, *n* (%)			
ER+, PR+, HER2−	10 (100%)	21 (100%)	31 (100%)

**Table 2:** Test Set Classification Performance: Summary of classification performance on the leave-one-out samples based on the nested leave-one-out cross-validation (LOOCV) technique. A 4-feature SVM-RBF model (*) generalized with 86% recall, 100% specificity, 93% balanced accuracy, an AUROC of 0.95 (CI: 0.88–1.00), and an AUPRC of 0.98 (CI: 0.94–1.00).

Classifier	Recall (%) (CI)	Specificity (%) (CI)	Accuracy (%) (CI)	Balanced Accuracy (%) (CI)	Precision (%) (CI)	NPV (%) (CI)	*F*1-*Score* (%) (CI)	AUROC (CI)	AUPRC (CI)
**LDA**	67	50	61	58	74	42	70	0.67	0.82
	(14/21)	(5/10)	(19/31)		(14/19)	(5/12)			
	(50–83)	(32–68)	(44–78)	(41–76)	(58–89)	(24–59)	(54–86)	(0.47–0.87)	(0.67–0.97)
**KNN *k* = 5**	71	70	71	71	83	54	77	0.78	0.85
	(15/21)	(7/10)	(22/31)		(15/18)	(7/13)			
	(56–87)	(54–86)	(55–87)	(55–87)	(70–96)	(36–71)	(62–92)	(0.62–0.94)	(0.72–0.99)
**SVM Linear**	71	60	68	66	79	50	75	0.54	0.76
	(15/21)	(6/10)	(21/31)		(15/19)	(6/12)			
	(56–87)	(43–77)	(51–84)	(49–82)	(65–93)	(32–68)	(60–90)	(0.32–0.76)	(0.59–0.93)
SVM-RBF ^(^*^)^	86	100	90	93	100	77	92	0.95	0.98
	(18/21)	(10/10)	(28/31)		(18/18)	(10/13)			
	(73–98)	(100–100)	(80–100)	(84–100)	(100–100)	(62–92)	(83–100)	(0.88–1.00)	(0.94–1.00)
**RF**	67	40	58	53	70	36	68	0.55	0.76
	(14/21)	(4/10)	(18/31)		(14/20)	(4/11)			
	(50–83)	(23–57)	(41–75)	(36–71)	(54–86)	(19–53)	(52–85)	(0.34–0.77)	(0.59–0.93)

NPV: Negative predictive value. AUROC: Area under the receiver operating characteristic curve. AUPRC: Area under the precision–recall curve. LDA: Linear discriminant analysis. SVM: Support vector machine. SVM-RBF: Support vector machine-radial basis function. RF: Random forest. CI: 95% Confidence interval.

## Data Availability

The datasets used and/or analyzed during the current study are available from the corresponding author on reasonable request.

## Abbreviations

The following abbreviations are used in this manuscript:

QUS	Quantitative ultrasound
ODXRS	Oncotype DX recurrence score
IQR	Inter-quartile range
LOOCV	Leave-one-out cross-validation
SVM-RBF	Support vector machine–radial basis function
AUROC	Area under the receiver operating characteristic curve
CI	Confidence interval
HR	Hormone receptor
HER2	Human epidermal growth factor receptor 2
LN	Lymph node
DRFS	Distant recurrence-free survival
RT	Radiation therapy
US RF	Ultrasound radio-frequency
CT	Computerized tomography
MRI	Magnetic resonance imaging
PET	Positron emission tomography
ER	Estrogen receptor
MBF	Mid-band fit
SS	Spectral slope
SI	Spectral intercept
ASD	Average scattering diameter
AAC	Average acoustic concentration
GLCM	Gray-level co-occurrence matrix
GLRM	Gray-level run-length matrix
GLSZM	Gray-level size zone matrix
NGTDM	Neighboring gray tone difference matrix
GLDM	Gray-level dependence matrix
MRMR	Maximal relevance minimal redundancy
SMOTE	Synthetic minority oversampling technique
SFS	Sequential feature selection
LDA	Linear discriminant analysis
KNN	*k*-nearest neighbors
RF	Random forest
PPV	Positive predictive value
NPV	Negative predictive value
AUPRC	Area under the precision–recall curve
IDC	Invasive ductal carcinoma
ILC	Invasive lobular carcinoma
DCIS	Ductal carcinoma in situ
FOV	Field-of-view
IDM	Inverse difference moment
CNN	Convolutional neural network
VIT	Vision transformer

## Appendix A

**Table A1:** US System Parameters: Physical parameters and acoustic beam characteristics from the L14-5/60 transducer on a Sonix Touch US system [13].

Parameters	Values
Number of elements	128
Kerf width [µm]	25
Element width [µm]	477
Elevation [mm]	4
Elevation focus [mm]	14
Depth of focus [mm]	16.7
Center frequency [MHz]	6.3
Bandwidth [MHz]	3–8
f\#	1.82
Axial resolution at 15 mm (−6 dB) [µm]	198
Lateral resolution at 15 mm (−6 dB) [µm]	483

**Table A2:** Radiomics features.

Feature Class	Feature Name
Morphological Features (*n* = 9)	Mesh Surface
	Pixel Surface
	Perimeter
	Perimeter-to-Surface Ratio
	Sphericity
	Spherical Disproportion
	Maximum 2D Diameter
	Major Axis Length
	Minor Axis Length
	Elongation
First-Order Statistical Features (*n* = 18)	10th Percentile
	90th Percentile
	Energy
	Entropy
	Interquartile Range
	Kurtosis
	Maximum
	Mean Absolute Deviation (MAD)
	Mean
	Median
	Minimum
	Range
	Robust Mean Absolute Deviation (rMAD)
	Root Mean Squared (RMS)
	Skewness
	Total Energy
	Uniformity
	Variance
GLCM (*n* = 24)	Autocorrelation
	Cluster Prominence
	Cluster Shade
	Cluster Tendency
	Contrast
	Correlation
	Difference Average
	Difference Entropy
	Difference Variance
	Inverse Difference (ID)
	Inverse Difference Moment (IDM)
	Inverse Difference Moment Normalized (IDMN)
	Inverse Difference Normalized (IDN)
	Informational Measure of Correlation (IMC) 1
	Informational Measure of Correlation (IMC) 2
	Inverse Variance
	Joint Average
	Joint Energy
	Joint Entropy
	Maximal Correlation Coefficient (MCC)
	Maximum Probability
	Sum Average
	Sum Entropy
	Sum Squares
GRLM (*n* = 16)	Gray-Level Nonuniformity
	Gray-Level Nonuniformity Normalized
	Gray-Level Variance
	High Gray-Level Run Emphasis
	Long Run Emphasis
	Long Run High Gray-Level Emphasis
	Long Run Low Gray-Level Emphasis
	Low Gray-Level Run Emphasis
	Run Entropy
	Run Length Nonuniformity
	Run Length Nonuniformity Normalized
	Run Percentage
	Run Variance
	Short Run Emphasis
	Short Run High Gray-Level Emphasis
	Short Run Low Gray-Level Emphasis
GLSZM (*n* = 16)	Gray-Level Nonuniformity
	Gray-Level Nonuniformity Normalized
	Gray-Level Variance
	High Gray-Level Zone Emphasis
	Large Area Emphasis
	Large Area High Gray-Level Emphasis
	Large Area Low Gray-Level Emphasis
	Low Gray-Level Zone Emphasis
	Size Zone Nonuniformity
	Size Zone Nonuniformity Normalized
	Small Area Emphasis
	Small Area High Gray-Level Emphasis
	Small Area Low Gray-Level Emphasis
	Zone Entropy
	Zone Percentage
	Zone Variance
GLDM (*n* = 14)	Dependence Entropy
	Dependence Nonuniformity
	Dependence Nonuniformity Normalized
	Dependence Variance
	Gray-Level Nonuniformity
	Gray-Level Variance
	High Gray-Level Emphasis
	Large Dependence Emphasis
	Large Dependence High Gray-Level Emphasis
	Large Dependence Low Gray-Level Emphasis
	Low Gray-Level Emphasis
	Small Dependence Emphasis
	Small Dependence High Gray-Level Emphasis
	Small Dependence Low Gray-Level Emphasis
NGTDM (*n* = 5)	Busyness
	Coarseness
	Complexity
	Contrast
	Strength

GLCM: Gray-level co-occurrence matrix. GRLM: Gray-level run-length matrix. GLSZM: Gray-level size-zone matrix. GLDM: Gray-level dependence matrix. NGTDM: Neighboring gray-tone difference matrix.

**Table A3:** Classifiers’ Hyperparameters: The machine learning classifiers and their hyperparameters (type and range of values to search).

Classifier	Hyperparameters	Values
SVM-Linear	C	{1 × 10^−4^, 1 × 10^−3^, 1 × 10^−2^, 1 × 10^−1^, 1, 10}
SVM-RBF	C	{1 × 10^−4^, 1 × 10^−3^, 1 × 10^−2^, 1 × 10^−1^, 1, 10}
	γ	{1 × 10^−3^, 1 × 10^−2^, 1 × 10^−1^, 1, 10, 100, 1000}
RF	N Estimators	{5, 10, …, 25}
	Criterion	{‘gini’, ‘entropy’}
	Max Tree Depth	{3, 4}
	Max Features	{‘sqrt’, ‘log2’}
	Max Samples	{0.5, 0.75, 0.9}

SVM: Support vector machine. SVM-RBF: Support vector machine–radial basis function. RF: Random Forest.
